# An investigation on the prevalence and patterns of multi-morbidity among a group of slum-dwelling older women of Kolkata, India

**DOI:** 10.1186/s12877-024-05078-y

**Published:** 2024-07-22

**Authors:** Anushka Ghosh, Susmita Mukhopadhyay, Barun Mukhopadhyay

**Affiliations:** Biological Anthropology Unit, Indian Statistical Institute, Kolkata, West Bengal India

**Keywords:** Older women, Health, Developing country, Slum, Correspondence analysis, Bi-plot

## Abstract

**Background:**

Multi-morbidity is a pervasive and growing issue worldwide. The prevalence of multi-morbidity varies across different populations and settings, but it is particularly common among older adults. It poses substantial physical, psychological, and socio-economic burdens on individuals, caregivers and healthcare systems. In this context, the present study aims to provide an insight on the prevalence and degree of multi-morbidity; and also, on the relationship between level of multi-morbidity and morbid conditions among a group of slum-dwelling older women.

**Methods:**

This community based cross-sectional study was conducted in the slum areas of urban Kolkata, West Bengal, India. It includes total 500 older women, aged 60 years or above. Pre-tested schedules on so-demographic and morbidity profile have canvassed to obtain the information by door-to-door survey. To determine the relationship between the level of multi-morbidity and morbid conditions, correspondence analysis has performed.

**Results:**

The study revealed three most prevalent morbid conditions- back and/or joint pain, dental caries/cavity and hypertension. The overall prevalence of multi-morbidity was 95.8% in this group of older women. It was highly over-represented by the oldest-old age group (80 years and above). Majority were found to suffer from five simultaneous morbid conditions that accounted for 15.2% of the total respondents. All of the oldest-old women of this study reported to suffer from more than two medical conditions simultaneously. Three distinct groups were formed based on the inter-relationship between level of multi-morbidity and morbid conditions. The group 1 and 2 represents only 27.8% and 18% of the total sample. Whereas, group 3 comprises the highest level of morbidities (≥ 6) and 52.8% of total sample, and strongly related with general debilities, cardiac problems, asthma/COPD, gastrointestinal, musculoskeletal problems, neurological disorders, hypothyroidism and oral health issues.

**Conclusion:**

The findings confirmed the assertion that multi-morbidity in slum living older adults is a problem with high prevalence and complexity. This study proposes an easily replicable approach of understanding complex interaction of morbidities that can help further in identifying the healthcare needs of older adults to provide them with healthy and more productive life expectancy.

**Supplementary Information:**

The online version contains supplementary material available at 10.1186/s12877-024-05078-y.

## Background

 Over the past few decades, every nation across the world has experienced demographic and epidemiologic transitions, primarily driven by population dynamics with notable increase in the presence of older individuals. One of the major consequences of this changing population age structure is that it imposes a greater strain on the health infrastructure of any nation because of the accentuated health complications with ageing [1, 2]. Health in terms of morbidity and disease aetiology grows more complicated when people acquire a greater number of ailments as their life expectancy increases [3, 4]. Therefore, geriatric health is no longer a matter of infectious versus non-communicable or acute versus chronic diseases; rather more likely of co-occurrence of diseases in a single individual. The simultaneous occurrence of two or more medical conditions without reference to any index condition is referred to as *multi-morbidity* [5, 6].

Multi-morbidity has been a recent construct of research, especially for geriatric populations. In this context, some literatures have highlighted that the intensity of disease co-occurrence has increased many folds among the older adults because of the age-related multisystem functional decline and progressive loss of resilience [7, 8]. The prevalence of multi-morbidity varies widely, ranging from 55 to 98% in most of the studies from developed countries [9]. But available literatures are limited that studied multi-morbidity in developing countries. A few studies conducted in India observed that on average elderly persons are reported with three simultaneous morbid conditions, and its prevalence ranged from 42.6 to 83% among different study populations [10, 11]. Also, multi-morbidity is a serious matter of concern since it leads to poor quality of life [12, 13], functional disability [14–16], frailty [17] and even premature death [7, 18].

In addition to physiological changes, a number of risk factors, from socio-economic to environmental and lifestyle, are associated with the development of multi-morbidity in old age. For instance, few studies from Australia and European countries have pointed out to the association of multi-morbidity with gender and socio-economic status [9, 19, 20]. Similarly, evidence based on National Sample Survey Organization (NSSO 2017-18) have indicated a higher prevalence of multi-morbidity among females and urban living older adults in India [21]. Likewise, lower socio-economic status, low education and living alone are also considered as risk factors for multi-morbidity; specially affecting the psychological health conditions [9, 20, 22]. Some other studies identified lifestyle behaviours as a predisposing risk factor. The factors like physical inactivity, smoking and high body mass index found to increase the risk of multi-morbidity [7, 23, 24]. But the findings are inconsistent and controversial as it remains less explored till now.

Multi-morbidity imposes a major strain on existing health infrastructure due to increasing demand for complex healthcare needs and practice of polypharmacy [25]. Unfortunately, despite this explosion of consequences related with multi-morbidity, the clinical guidelines and healthcare practices are still primarily centred around *mono-morbid* conditions, especially in the developing countries [2]. Thus, in the present context of global ageing, it is imperative to shift this approach towards multi-morbidity studies. Indeed, the pattern of disease clustering and their interrelationships required to be specially emphasized to obtain wider understanding and ensure *healthy* life expectancy for all.

The aim of this study is to provide relevant insight on the prevalence of overall morbidity status and degree of simultaneous co-occurrence of these conditions in a group of slum-living older women. Furthermore, we also attempt to understand intricate inter-dependent relationship between degree of multi-morbidity and morbid conditions along with the identification of strongly associated conditions.

## Methods

### Participants

This community based cross-sectional study on the health aspects of slum-dwelling older persons was supported by Indian Statistical Institute, Kolkata, India. This study was conducted on the urban slum areas of Kolkata Municipal Corporation (KMC), West Bengal, India. Initially, five KMC wards (number 7, 8, 15, 27 and 28) were purposively selected based on operational feasibility. Subsequently, a complete enumeration methodology was implemented to encompass the maximum number of study participants from each ward. A roster was compiled utilizing the latest updated electoral register of the designated areas and each participant was individually approached at their domicile to obtain consent for data collection. Data on the morbidity profile of the participants had been collected between February and April 2019 by direct interview method.

The study comprised 500 women aged 60 years and above, considering the prevailing predominance of females in the geriatric cohort worldwide, and underscored by persistent disparities in resource allocation within households, causing an increased burden of poverty among this population subset. The study was restricted to Hindu older women only to avoid possible ethnic and lifestyle related differences. Institutional ethical approval and informed consent from participants (other than non-literates) had obtained prior to data collection. The participants who were unable to read and write, the informed consent was obtained from a legal guardian or caregivers.

### Data

Pre-designed and pre-tested schedules were canvased to record information on socio-demographic condition and morbidity profile of the participants by door-to-door survey, approaching participants at their residence.

### Socio-demographic characteristics

Socio-demographic characteristics include age of the participant at the time of interview (in completed years), educational status, occupational status, marital status and monthly per-capita expenditure (in INR). Age of the participants was further divided into three groups where elderly aged 60 to 69 years were categorized as young-old, 70 to 79 years were categorized as old-old and age of 80 years and above as oldest-old group. Educational attainment of the participants comprises five categories such as non-literate, literate, primary, secondary and undergraduate/graduate level. Occupational status comprises those who are working (i.e. gainfully employed) or non-working (i.e. unemployed). Per-capita monthly expenditure was divided into three equal groups following the tertile division.

### Morbidity profile

Morbidity profile of the study participants comprises both morbidity symptoms and diagnosed or clinically evaluated conditions present at the time of data collection. The reported diagnosed morbid conditions were further cross verified with laboratory test reports and prescriptions provided by trained medical practitioners. A total of 50 morbidities was documented in this study which was perhaps categorized into fifteen different domains based on the topographic classification of diseases i.e. based on the relatedness to bodily systems. These included domains were general debility, eye problem, ear-nose-throat (ENT), cardiovascular, respiratory, gastrointestinal, urinary-renal, musculoskeletal-rheumatologic, dermatological, neurological, psychological, endocrinal, hematologic-lymphatic and oral health. Additionally, three uncategorized morbidities were also included in a separate domain (named as *other*).

Multi-morbidity was defined as the co-existence of two or more morbid conditions at a time. The simultaneous occurrence of two or more illnesses among fifty reported medical conditions explained the degree of multi-morbidity. Accordingly, level ‘0’ indicates participants had no health complications reported at the time of interview; level ‘1’ indicates those suffer from one medical condition; and presence of more than one morbidity indicates the ‘multi-morbid’ condition.

### Statistical analysis

Description of the variables was provided in terms of total counts, relative frequencies and mean. The prevalence of the morbid conditions was estimated based on the proportion of positive cases among total study sample.

### Bi-variate statistics

Chi-square test of association was conducted to show the relationship between morbid conditions and age cohorts. Fisher’s exact test was performed instead of chi-square test where any cell of the contingency table contains a value of less than five. A *p*-value of less than 0.05 was considered significant in all cases.

### Correspondence analysis (CA)

To determine the interdependent relationship between the level of multi-morbidity and morbid conditions, CA was conducted. It is a non-parametric multivariate exploratory analysis that attempts to identify the proximal relationship between variables. In this analysis, multi-morbidity level (categorized from 1 to > 6) was considered as row profile and morbid conditions (total 50 variables represented the morbid conditions) were considered as column profile. Symmetrical normalization method was used to standardize row and column data. It represents the variable categories in a multi-dimensional space and the number of dimensions was estimated by subtracting one from the total number of variables in a set that contains the least number of variables. In our data set, multi-morbidity level consists least number of variables (7), so 6 dimensions were extracted here which were arranged in ascending order based on the amount of variance explained in the model. Chi-square statistic was applied to test for total variance explained along with the associated probability. A probability value of less than 0.05 was considered to test significance of the model. Furthermore, it calculated canonical correlation between variables - multi-morbidity and medical conditions. Squaring the value of canonical correlation, the inertia or variance was derived; total inertia indicates the total amount of variance accounted for each multi-morbidity level or each morbid condition, whereas proportion of inertia provides the percent of variance that each dimension explains out of the total variance. Relative precision (confidence singular value) and correlation of the dimensions was also identified.

The overview of row and column points allow to evaluate the contribution of each multi-morbidity level and morbid condition to the dimensions, respectively. This analysis evaluates the loading of each row/column points as well as explains the degree of extraction of dimensions with each point. It offers a concise view regarding the larger contribution of specific multi-morbidity level and morbid conditions in the study population.

#### Bi-plot

The most relevant is the graphical presentation of data matrix (or contingency table) provided that helps to visualize the relationships among categories spatially on dimensional axes. Each row and Column were depicted as a single point in a bi-plot (a type of scatter plot) that reveals association or similarities between variable categories through their proximity. Thus, the points that were mapped close to one another in the bi-plot share similar profiles; and those mapped far away from one another have very different profiles. Additionally, the distance between each row variable with each of the column variable was measured using the following formula:

$$\mathrm{Distance}=\sqrt{\left[\left(x_2-x_1\right)^2+\left(y_2-y_1\right)^2\right]}$$

Here ${x}_{1}$ and ${y}_{1}$ are the row points (multi-morbidity levels); and ${x}_{2}$ and ${y}_{2}$ are column points (morbid conditions). A lower value of distance indicates close correspondence between each row point (multi-morbidity level) and column point (morbid condition).

Afterwards, we have identified three groups by inspecting the coordinates/position of each multi-morbidity level in the bi-plot. Group 1 contributed to the positive side of both dimension 1 and 2; group 2 contributed to positive and negative side of only one dimension; and group 3 contributed to negative side of both dimensions. Finally, the correspondence between variables in each group was identified based on two principles viz. (a) the lowest value of distance of each morbid condition across multi-morbidity levels (horizontally), and (b) the lowest value of distance of each level of multi-morbidity across all morbid conditions (vertically).

Data analysis was performed on a statistical package PASW (Predictive Analytics Software) version 18.0 [26].

## Results

Table 1 shows majority (64.4%) of the participants were aged between 60 and 69 years that represents young-old age group and was followed by old-old age group (25.2%). Among the total population studied, 24.6% were living in wedlock while 72.8% belong to W/D/S group (among them 71.8% were widow). Educational status showed 50.4% of the participants did not have any formal education. Most of the participants (67.8%) were unemployed and only 32.2% were gainfully employed in mainly unorganized sector. The lowest range of the monthly per-capita expenditure was INR 1,428 (approx.) and 66% of the participants had per-capita expenditure below INR 2,000.

**Table 1:** Baseline information of the study participants

Socio-demographic characteristics	**Total %** ***n* = 500**
***Age groups***	
Young old (60–69 years)	322 (64.4)
Old-old (70–79 years)	126 (25.2)
Oldest-old (≥ 80 years)	52 (10.4)
***Marital status***	
Married	123 (24.6)
Unmarried	13 (2.6)
W/D/S^a^	364 (72.8)
***Educational status***	
Non-literate	252 (50.4)
Literate	74 (14.8)
Primary	58 (11.6)
Secondary	113 (22.6)
Undergraduate/Graduate	3 (0.6)
***Occupational status***	
Gainfully employed (Working)	161 (32.2)
Unemployed (Non-working)	339 (67.8)
***Monthly per-capita expenditure (in INR)***	
≤ 1428.57	163 (32.6)
1428.58–2000	167 (33.4)
> 2000	170 (34.0)

^a^*W/D/S* Widow/Divorcee/Separated, *INR* Indian Rupee

### Prevalence of morbidities

Table 2 summarizes the morbidity profile of respondents as well as the distribution of morbid conditions throughout the age cohorts. It was found that majority of the respondents suffered from back and/or joint pain, dental caries/cavity and hypertension with an overall prevalence of 83.6%, 73.2% and 49.2%, respectively. Less number of respondents (with < 1% prevalence rate) were diagnosed with the conditions such as peripheral edema, liver cirrhosis, stomach ulcer, gallstone, sciatica, hypoglycemia, allergy and herpes (0.2%); anorexia, cerebral atrophy, anemia and depression/anxiety (0.4%); sinusitis, tonsillitis/throat infection, UTI, headache/migraine, cancer and hernia (0.6%); hemorrhoid and renal failure (0.8%).

**Table 2:** Overall and age group specific prevalence of morbid conditions

**Morbid conditions**	**Total %** ***n*=500**	**Young-old (%)** ***n*=322 (64.4)**	**Old-old (%)** ***n*=126 (25.2)**	**Oldest-old (%)** ***n*=52 (10.4)**	***P*-value**
***General debility***					
M1. Anorexia	2 (0.4)	1 (0.3)	1 (0.8)	-	-
M2. Persistent fever/ Common cold	160 (32.0)	107 (33.2)	40 (31.7)	13 (25.0)	0.497
M3. Vitamin/Mineral deficiency^a^	36 (7.2)	20 (6.2)	11 (8.7)	5 (9.6)	0.462
***Eye***					
M4. Vision loss	22 (4.4)	9 (2.8)	8 (6.3)	5 (9.6)	0.039
M5. Cataract^a^	37 (7.4)	18 (5.6)	15 (11.9)	4 (7.7)	0.078
***ENT***					
M6. Hearing loss	150 (30.0)	75 (23.3)	46 (36.5)	29 (55.8)	0
M7. Sinusitis^a^	3 (0.6)	2 (0.6)	1 (0.8)	-	-
M8. Tonsillitis/Throat infection^a^	3 (0.6)	3 (0.9)	-	-	-
***Cardiovascular***					
M9. Peripheral edema^a^	1 (0.2)	1 (0.3)	-	-	-
M10. Hypertension^a^	246 (49.2)	144 (44.7)	68 (54.0)	34 (65.4)	0.01
M11. Hypercholesterolemia^a^	9 (1.8)	7 (2.2)	2 (1.6)	-	-
M12. Coronary heart disease/ Arrhythmia/Stroke/Chest pain/Palpitation^a^	49 (9.8)	25 (7.8)	18 (14.3)	6 (11.5)	0.102
***Respiratory***					
M13. Asthma/Chronic Obstructive Pulmonary Disease (COPD)^a^	34 (6.8)	18 (5.6)	10 (7.9)	6 (11.5)	0.241
***Gastrointestinal***					
M14. Indigestion/Flatulence/Abdominal pain/Nausea/Vomiting	141 (28.2)	77 (23.9)	43 (34.1)	21 (40.4)	0.012
M15. Constipation / Bloody stools	10 (2.0)	7 (2.2)	2 (1.6)	1 (1.9)	1
M16. Acid reflux	85 (17.0)	54 (16.8)	22 (17.5)	9 (17.3)	0.983
M17. Irregular bowel habit	23 (4.6)	12 (3.7)	9 (7.1)	2 (3.8)	0.305
M18. Gastritis^a^	129 (25.8)	72 (22.4)	39 (31.0)	18 (34.6)	0.054
M19. Hemorrhiods^a^/Anal fissure^a^	4 (0.8)	3 (0.9)	1 (0.8)	-	-
M20. Liver cirrhosis^a^	1 (0.2)	-	1 (0.8)	-	-
M21. Stomach ulcer^a^	1 (0.2)	-	1 (0.8)	-	-
M22. Gallstone^a^	1 (0.2)	-	-	1 (1.9)	-
***Urinary & Renal***					
M23. Urinary incontinence	177 (35.4)	11 (34.2)	45 (35.7)	22 (42.3)	0.52
M24. Urinary Tract Infection (UTI)^a^	3 (0.6)	1 (0.3)	1 (0.8)	1 (1.9)	0.181
M25. Renal failure^a^	4 (0.8)	3 (0.9)	-	1 (1.9)	-
***Musculoskeletal & Rheumatologic***					
M26. Back / Joint pain	418 (83.6)	286 (83.2)	107(84.9)	43 (82.7)	0.894
M27. Joint swelling	235 (47.0)	152 (47.2)	57 (45.2)	26 (50.0)	0.839
M28. Spondylosis^a^	9 (1.8)	8 (2.5)	-	1 (1.9)	-
M29. Arthritis^a^	18 (3.6)	10 (3.1)	8 (6.3)	-	-
M30. Hyperuricemia^a^	6 (1.2)	4 (1.2)	1 (0.8)	1 (1.9)	0.664
***Dermatological***					
M31. Infection/Rash/Itching	8 (1.6)	5 (1.6)	2 (1.6)	1 (1.9)	0.876
M32. Skin lesion	13 (2.6)	10 (3.1)	2 (1.6)	1 (1.9)	0.827
***Neurological***					
M33. Dizziness	231 (46.2)	149 (46.3)	52 (41.3)	30 (57.7)	0.136
M34. Headache/Migraine^a^	3 (0.6)	2 (0.6)	1 (0.8)	-	-
M35. Tremors	17 (3.4)	9 (2.8)	7 (5.6)	1 (1.9)	0.33
M36. Insomnia^a^	21 (4.2)	8 (2.5)	8 (6.3)	5 (9.6)	0.022
M37. Sciatica^a^	1 (0.2)	-	1 (0.8)	-	-
M38. Cerebral atrophy^a^	2 (0.4)	1 (0.3)	1 (0.8)	-	-
***Psychological***					
M39. Depression/Anxiety^a^	2 (0.4)	2 (0.6)	-	-	-
***Endocrinal***					
M40. Diabetes mellitus^a^	54 (10.8)	36 (11.2)	11 (8.7)	7 (13.5)	0.609
M41. Hypothyroidism^a^	19 (3.8)	15 (4.7)	3 (2.4)	1 (1.9)	0.547
M42. Hypoglycaemia^a^	1 (0.2)	1 (0.3)	-	-	-
***Hematologic & lymphatic***					
M43. Anemia^a^	2 (0.4)	1 (0.3)	-	1 (1.9)	-
M44. Allergy^a^	1 (0.2)	1 (0.3)	-	-	-
***Oral***					
M45. Dental cavity/caries^a^	366 (73.2)	233 (72.4)	97 (77.0)	36 (69.2)	0.484
M46. Any infection / Toothache	50 (10.0)	38 (11.8)	9 (7.1)	3 (5.8)	0.222
M47. Oral ulcer^a^	54 (10.8)	36 (11.2)	13 (10.3)	5 (9.6)	0.926
***Others***					
M48. Cancer^a^	3 (0.6)	2 (0.6)	1 (0.8)	-	-
M49. Hernia^a^	3 (0.6)	2 (0.6)	1 (0.8)	-	-
M50. Herpes^a^	1 (0.2)	1 (0.3)	-	-	-

^a^Clinically diagnosed morbidity

It was also found that six out of the total fifty morbid conditions included in this study were significantly associated with age groups viz. severe vision loss, severe hearing loss, hypertension, indigestion, gastritis and insomnia (Table 2). All these morbid conditions along with vitamin/mineral deficiency, asthma/COPD, urinary incontinence and UTI were observed to increase with age. The prevalence of back/joint pain remains high across all age groups (Table 2).

**Fig. 1:**
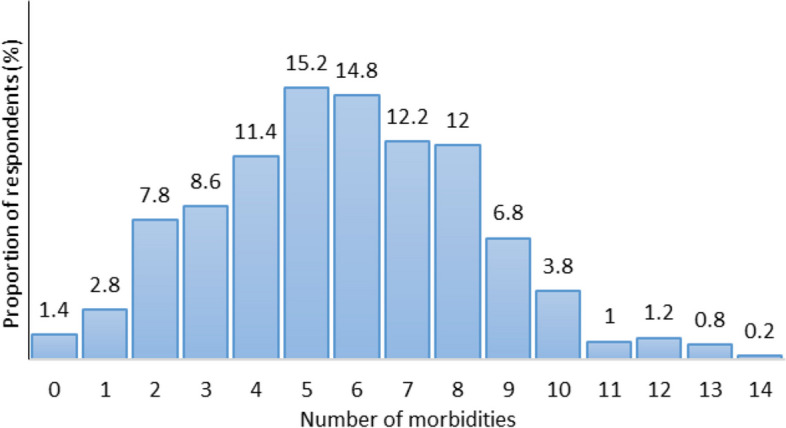
Distribution of population according to number of morbidities

### Level of multi-morbidity

Table 3 presents that the estimated prevalence of multi-morbidity in the study population was 95.8%. About 1.4% of the respondents were reported to have none of these illnesses and 2.8% reported with only one morbidity. Multi-morbidity was highly over-represented by the oldest-old age group. But no statistical significance was found between number of morbidity and age group. The overall magnitude of multi-morbidity ranged from 0 to as high as 14, where the mean and median is around 5.74 and 6, respectively. Majority of the respondents i.e. 15.2% and 14.8% found to have 5 and 6 simultaneous morbid conditions, respectively (Fig. 1).

**Table 3:** Prevalence of multi-morbidity in study population, by age cohorts

**No. of morbidities**	**Total %** ***n* = 500**	**Young-old %** ***n* = 322 (64.4)**	**Old-old %** ***n* = 126 (25.2)**	**Oldest-old %** ***n* = 52 (10.4)**	***P*-value**
0	7 (1.4)	5 (1.6)	2 (1.6)	-	(1.615) 0.810
1	14 (2.8)	10 (3.1)	4 (3.2)	-	
2	39 (7.8)	307 (95.3)	120 (95.2)	52 (100.0)	
3	43 (8.6)				
4	57 (11.4)				
5	76 (15.2)				
6	74 (14.8)				
7	61 (12.2)				
8	60 (12.0)				
9	34 (6.8)				
10	19 (3.8)				
11	5 (1.0)				
12	6 (1.2)				
13	4 (0.8)				
14	1 (0.2)				
**Mean ± SD**	**5.74 ± 2.61**				

### Inter-relationship between level of multi-morbidity and morbid conditions

Table 4 provides the summary information of the correspondence analysis. Chi-square statistic value (χ^2^ = 700.79, *p*-value = 0.000) revealed strong correspondence between number of morbidities and morbid conditions; and the model was highly significant. The value of canonical correlation (dimension 1 = 0.337 and dimension 2 = 0.242) and inertia (amount of variance) was greater for first two dimensions. A total of 23.8% variance (inertia) can be explained by all dimensions in the model. Dimension 1 explains 47.7% and dimension 2 explains 24.5% of total 23.8% of variance explained in the model. Dimension 3 explains only 9.1% of variance that reduced thereafter. Therefore, dimension 1 and 2 provides better understanding than others and thus considered for further interpretations (see table A1 and A2 in supplementary material). The confidence singular value reveals that the second dimension was more precise than the first and both the dimensions were highly correlated (0.661).

**Table 4:** Correspondence between the level of multi-morbidity and morbid conditions

**Dimension**	**Canonical correlation**	**Inertia**	**χ**^**2**^ **(*p*-value)**	**Proportion of Inertia**		**Confidence Singular Value**	
				**Accounted for**	**Cumulative**	**Standard Deviation**	**Correlation**
1	0.337	0.114	700.786 (0.000)	0.477	0.477	0.031	0.661
2	0.242	0.058		0.245	0.722	0.018	
3	0.147	0.022		0.091	0.812		
4	0.143	0.020		0.085	0.897		
5	0.124	0.015		0.065	0.962		
6	0.095	0.009		0.038	1.000		
Total		0.238		1.000	1.000		

**Fig. 2:**
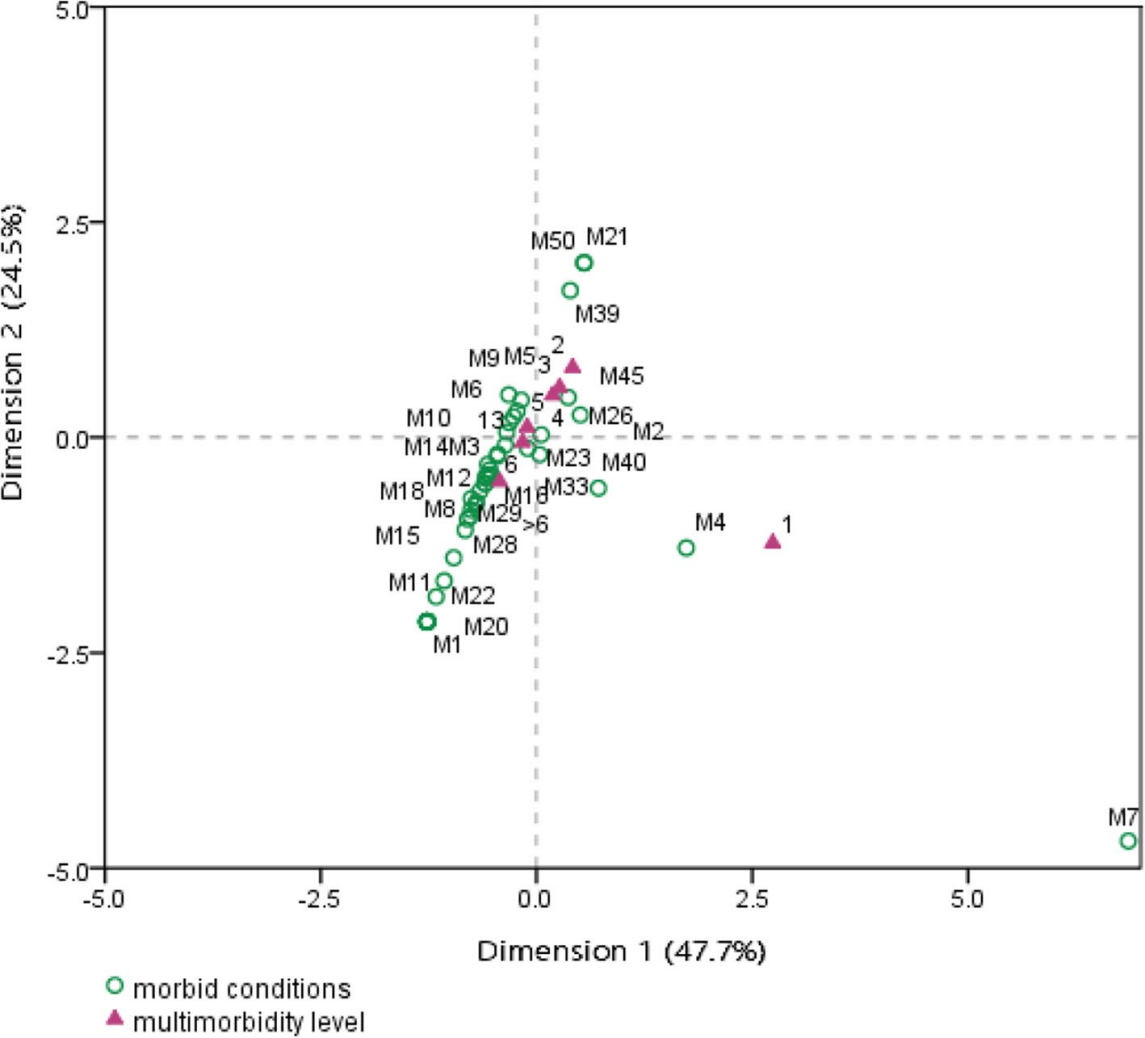
Bi-plot showing proximal distance between level of multi-morbidity and morbid conditions

Figure 2 is the visual representation of the relationship between level of multi-morbidity and reported morbid conditions. The distance between level of multi-morbidity with each morbid condition in this two dimensional bi-plot is presented in table A3 of supplementary material, which depicts the following:

- *Group 1* represents the positive side of both dimensions (1 and 2) that comprises lower levels of multi-morbidity such as 2, 3 and 4. Following the assumptions, the result reveals that multi-morbidity levels 2, 3 and 4 were strongly associated with morbid conditions such as back and/or joint pain, dental cavity/caries and depression/anxiety. This group comprises 27.8% of total sample. About 69.8% of the participants had reported with back and/or joint pain, 63.3% had dental cavity/caries and 0.7% had psychological disorder.
- Similarly, *Group 2* contributing to the positive side of dimension 2 (but negative side of dimension 1) and include the multi-morbidity level 5. The strongly corresponded morbid conditions with this level were cataract, hearing loss, hypertension, irregular bowel habit, urinary incontinence and peripheral edema. In opposition, morbidity level 1 was located on the negative side of dimension 2 (but positive side of dimension 1) and related to vision loss only. Group 2 comprises 18% of total sample. Hypertension accounted for 43.3% of the participants that followed by urinary incontinence with 27.8%, hearing loss with 20%, cataract with 7.8%, irregular bowel habit with 5.6% and peripheral edema with 1.1%. Whereas, 4.4% of the participants were reported to have vision loss.
- *Group 3* contributing to the negative side of both dimensions (1 and 2); and comprises the highest level of multi-morbidity, 6 and above. The higher multi-morbidity levels (6 and > 6) were strongly associated with general debilities (including persistent fever/common cold and vitamin/mineral deficiency), cardiac problems (incl. coronary heart disease/arrhythmia/stroke/chest pain/palpitation), asthma/COPD, gastrointestinal disorders (incl. indigestion, acid reflux and gastritis), musculoskeletal problems (incl. joint swelling and hyperuricemia), neurological disorders (incl. dizziness, headache/migraine and tremor), hypothyroidism and oral health issues (incl. infection/toothache and oral ulcer). This group comprises majority (52.8%) of total sample. About 64% of the participants in this group had joint swelling, 63.3% reported with dizziness, 46.6% with persistent fever/common cold, 43.2% with indigestion, 40.5% with gastritis, 26.1% with acid reflux, 17.4% with oral ulcer, 15.2% with heart problems, 14.8% with oral infection/toothache, 10.6% with vitamin/mineral deficiency, 10.2% with asthma/COPD, 6.1% with hypothyroidism, 4.2% with tremor, 1.9% with hyperuricemia and 1.1% with headache/migraine.

## Discussion

The findings obtained from this study generally highlighted the correspondence between the level of multi-morbidity and reported morbidities. The burden of persistent and simultaneous occurrence of morbidities has been identified as an obstacle of healthy ageing. For the developing countries like India, the risk of ageing with multi-morbidity is a major threat because of the infrastructural and economic inadequacies. Also owing to the socio-economic and ethnic variations within the country, it became imperative to provide special emphasis on the resource poor population groups like slum-dwellers, highlighting its significance as a public health concern.

In the slum-living geriatric study population, a total of fifty morbid conditions have been reported; of which back/joint pain was the most prevalent ailment found among 83.6% of the respondents; specially among young-old women, indicating early onset of this symptom with advancement of age. Higher prevalence of musculoskeletal disorders including joint pain and arthritis was also stated in many studies conducted both in urban slums and rural areas of India where the prevalence ranged from 46 to 70.4% and the severity was reflected more among the older women than men [11, 27, 28]. Furthermore, this study also corroborates with the evidence that highlighted higher occurrence of hypertension and oral problems among slum living older adults [27–29]. Furthermore, the study observed that psychological health issues remain neglected or undiagnosed; depression and anxiety were the only psychological disorders that was reported by 0.4% of older women. In another study, Ghosh and Mukhopadhyay (2021) have documented higher prevalence of depression, anxiety and cognitive decline on same population group of slum-living older women of Kolkata while canvassing geriatric psychological screening tests [30]. Comparing the prevalence of morbidities among different age groups, this study showed that conditions such as dual sensory loss (both vision and hearing loss), hypertension, indigestion, gastritis and insomnia differed significantly with age and observed more among oldest-old group. Likewise, few studies on slum and non-slum dwelling geriatric populations of India exhibited high incidence of disabilities especially among women aged more than 65 years, though the prevalence differ from one group to other [31, 32].

Only 1.4% of the study population has been demonstrated with no medical condition and 2.8% have suffered from one medical condition. A substantially higher proportion (95.8%) of the study population have diagnosed with multi-morbidity that defined as having co-existing two or more morbid conditions. Interestingly, the degree of multi-morbidity was as high as the co-occurrence of 14 conditions that specifies high risk of multi-morbidity among this group of older women. Additionally, it revealed that majority of the respondents, about 15.2%, sustained minimum of five different morbidities simultaneously. Other studies among different geriatric populations based on the similar definition of multi-morbidity showed a wider variation in prevalence rates. The prevalence of multi-morbidity in Korea (85.2%), Australia (75%), England (62.8%), Europe (55 to 73.25%) and Bangladesh (53.8%) were lower than the estimation of present study [5, 8–10, 33, 34]. Contradictorily, a study by Rocca et al. (2014) have documented lower prevalence of multi-morbidity in Asian countries. Studies based on larger population based surveys from India determined the states with higher prevalence of multi-morbidity that includes Kerala (24 to 42.02%), Punjab (6 to 35.78%), Maharashtra (23.42%), West Bengal (23.15%) etc. [14, 35].

A positive association between age and the degree of multi-morbidity was suggested in this study. The rate of multi-morbidity increased with increasing age; and all of the participants of oldest-old age group had two or more diseases. These findings corroborate with other studies where the authors suggest that the intensity of multi-morbidity was the highest among older aged above 75 years and the rate of prevalence varied from 33.97 to 98.5% in different countries across the world [4, 11, 24, 34, 36].

This study was also indicative of a significant correspondence between level of multi-morbidity and diseases. Based on their association, three distinct clusters were identified. First cluster or Group 1 comprised of elderly with comparatively low multi-morbidity and associated with medical conditions like back/joint pain, dental cavity/caries and depression/anxiety. Nearly two-third participants of this group reported back/joint pain and dental cavity/caries. Hence, the participants of this group showed less burden of morbidities than others with a proportion of 27.8% of total sample. Group 2 was characterized by the older with five co-exiting diseases viz. cataract, severe hearing loss, hypertension, irregular bowel habit, urinary incontinence and peripheral edema. This particular group or cluster consisted 18% of the sample where hypertension was the most prevalent that followed by urinary incontinence and severe hearing loss. Finally, Group 3 comprised the highest level of multi-morbidity; the highly prevalent cluster of morbidities associated with this group were swelling of joints, dizziness, persistent fever/common cold, indigestion, gastritis, acid reflux etc. This group was made up of 52.8% of the total sample. Therefore, this cluster has the highest burden of morbidities and co-occurrences of these particular conditions may largely responsible for deteriorating health of slum-dwelling older women. In accordance with the present findings, a study among hospitalized elderly patients in England has estimated the relatedness between multi-morbidity, chronic diseases and age groups where they identified the highest disease burden with six or more morbidities and its associated chronic conditions were heart failure, cerebrovascular accident, diabetes, hypertension and myocardial infarction [33]. Unlike the present study, Ruiz and colleagues (2015) have reported 22.2% of older patients were at highest risk of multi-morbidity.

The present study comprises some limitations that required to be addressed further. Firstly, because of the geographical restriction of the study within urban Kolkata, no generalization from the results could be drawn that represents the national population. At the same time, just as this study particularly highlighted the health conditions of more vulnerable older women, it is also essential to shed light on the problems of older men with the interest of improving overall health status of this age group. Another limitation of this study is its inability to offer a comparative analysis across diverse religious groups, as it exclusively focused on the Hindu population. Again, the cross-sectional design of this study was unable to determine the long-term consequences of multi-morbidity. Therefore, longitudinal studies will help to understand the complex disease interactions with time. Another limitation of this study in assessing the prevalence of diseases is attributed to under-diagnosis and under-reporting of many conditions due to their inappropriate health-seeking behaviours and lack of awareness. Nevertheless, this study has its strength in investigating a wide range of clinically evaluated morbidities among a marginalized group in India.

## Conclusion

All these findings have confirmed the assertion that multi-morbidity in older adults is a problem with high prevalence and provides an overview on association of certain ailments with different levels of multi-morbidity. It is a complex phenomenon to encounter and most significant to understand complex disease aetiology where severity increased with age. In developing countries, the older inhabitants of resource poor areas are at the edge of threat mainly because of their stressful livelihood. The burden of persistent and simultaneous occurrence of diseases is one of the main barriers of healthy ageing for them. This study has identified the complex inter-relationship of different morbid conditions with increasing the level and prevalence of multi-morbidity. The dynamics of multi-morbidity related with impecunious older women, is a serious issue that demands attention from healthcare providers, researchers, policymakers and society as a whole to develop strategies and favourable healthcare models. Besides, the challenges posed by multi-morbidity necessitate a shift from the traditional disease-focused model of healthcare to a more holistic and patient-centred approach. Integrated care models, multidisciplinary collaboration and the use of digital health technologies have shown promise in improving outcomes for individuals with multi-morbidity in present day scenario. This study may be competent to propose an easily replicable approach of understanding multifaceted relationship between multi-morbidity and diseases for future investigations. However, there is still a need for continued research efforts to introduce innovative strategies to optimize care delivery and support the effective management of multi-morbidity for achieving healthier and more resilient ageing communities.

### Supplementary Information

Supplementary Material 1.

## Data Availability

All data analysed during this study can be obtained from the corresponding author upon reasonable request.
