# Supplementary material for: An investigation on the prevalence and patterns of multi-morbidity among a group of slum-dwelling older women of Kolkata, India
Source: BMC Geriatr. 2024 Jul 22;24:624. doi: 10.1186/s12877-024-05078-y (PMC11265169; doi:10.1186/s12877-024-05078-y)
Supplement: Supplementary file 1 — Supplementary Material 1. [file 12877_2024_5078_MOESM1_ESM.docx]

**Supplementary material**

**Table A1.** Overview of row points across multi-morbidity scores

| **Multi-morbidity** | **Mass** | **Score in Dimension** | | **Inertia** | **Contribution of point to Inertia of Dimension** | | **Contribution of dimension to Inertia of Point** | | **Total** |
| --- | --- | --- | --- | --- | --- | --- | --- | --- | --- |
|  |  | **1** | **2** |  |  |  |  |  |  |
|  |  |  |  |  | **1** | **2** | **1** | **2** |  |
| **1** | 0.034 | 2.736 | -1.227 | 0.098 | 0.753 | 0.211 | 0.874 | 0.126 | 1.000 |
| **2** | 0.068 | 0.417 | 0.810 | 0.027 | 0.035 | 0.185 | 0.149 | 0.403 | 0.552 |
| **3** | 0.102 | 0.267 | 0.577 | 0.018 | 0.022 | 0.141 | 0.133 | 0.445 | 0.578 |
| **4** | 0.136 | 0.186 | 0.489 | 0.024 | 0.014 | 0.135 | 0.065 | 0.323 | 0.388 |
| **5** | 0.170 | -0.109 | 0.119 | 0.013 | 0.006 | 0.010 | 0.050 | 0.044 | 0.094 |
| **6** | 0.204 | -0.162 | -0.058 | 0.017 | 0.016 | 0.003 | 0.108 | 0.010 | 0.118 |
| **>6** | 0.286 | -0.427 | -0.516 | 0.041 | 0.155 | 0.315 | 0.432 | 0.451 | 0.882 |
| **Active Total** | 1.000 |  |  | 0.238 | 1.000 | 1.000 |  |  |  |

The participants with multi-morbidity level of more than 6 showed the highest proportion of positive responses (0.286 or 28.6%) with respect to morbid conditions. The scores in dimensions revealed that multi-morbidity level 1 (9.8%) and level >6 (4.1%) independently consisted the highest proportion of variance. The contribution column reflects that multi-morbidity 1 loads heavily on dimension 1 (75.3%) but not on dimension 2 (21.1%). It can also be seen that extraction of dimension 1 explains 87.4% of variance whereas dimension 2 explains only 12.6% of variance. Again, multi-morbidity >6 loads heavily on dimension 2 (31.5%) and explain 45.1% of variance (dimension 2) across morbid conditions.

| **Table A2.** Overview of column points across morbid conditions | | | | | | | | | |
| --- | --- | --- | --- | --- | --- | --- | --- | --- | --- |
| **Morbid conditions** | **Mass** | **Score in dimension** | | **Inertia** | **Contribution of point to inertia of dimension** | | **Contribution of dimension to inertia of point** | | **Total** |
|  |  |  |  |  |  |  |  |  |  |
|  |  | **1** | **2** |  | **1** | **2** | **1** | **2** |  |
| **M1** | 0.000 | -1.268 | -2.137 | 0.001 | 0.002 | 0.007 | 0.217 | 0.441 | 0.658 |
| **M2** | 0.054 | 0.037 | -0.202 | 0.002 | 0.000 | 0.009 | 0.013 | 0.280 | 0.293 |
| **M3** | 0.010 | -0.542 | -0.377 | 0.003 | 0.009 | 0.006 | 0.298 | 0.103 | 0.401 |
| **M4** | 0.010 | 1.736 | -1.280 | **0.016** | 0.086 | 0.066 | 0.630 | 0.245 | 0.875 |
| **M5** | 0.013 | -0.177 | 0.432 | 0.002 | 0.001 | 0.010 | 0.074 | 0.318 | 0.393 |
| **M6** | 0.052 | -0.223 | 0.304 | 0.003 | 0.008 | 0.020 | 0.342 | 0.454 | 0.796 |
| **M7** | 0.003 | 6.857 | -4.685 | **0.059** | **0.389** | 0.253 | **0.749** | 0.250 | 0.999 |
| **M8** | 0.001 | -0.827 | -1.075 | 0.001 | 0.002 | 0.004 | 0.190 | 0.230 | 0.420 |
| **M9** | 0.000 | -0.322 | 0.494 | 0.002 | 0.000 | 0.000 | 0.007 | 0.012 | 0.019 |
| **M10** | 0.085 | -0.265 | 0.239 | 0.004 | 0.018 | 0.020 | 0.485 | 0.282 | 0.767 |
| **M11** | 0.002 | -1.071 | -1.663 | 0.002 | 0.006 | 0.022 | 0.303 | 0.524 | 0.827 |
| **M12** | 0.014 | -0.579 | -0.469 | 0.003 | 0.014 | 0.013 | 0.495 | 0.233 | 0.728 |
| **M13** | 0.010 | -0.449 | -0.195 | 0.001 | 0.006 | 0.002 | 0.553 | 0.075 | 0.628 |
| **M14** | 0.040 | -0.560 | -0.435 | 0.007 | 0.037 | 0.031 | 0.589 | 0.255 | 0.843 |
| **M15** | 0.002 | -0.960 | -1.395 | 0.002 | 0.006 | 0.019 | 0.296 | 0.449 | 0.745 |
| **M16** | 0.026 | -0.455 | -0.220 | 0.003 | 0.016 | 0.005 | 0.584 | 0.098 | 0.681 |
| **M17** | 0.007 | -0.313 | 0.170 | 0.004 | 0.002 | 0.001 | 0.065 | 0.014 | 0.078 |
| **M18** | 0.036 | -0.658 | -0.628 | 0.011 | 0.046 | 0.059 | 0.466 | 0.304 | 0.770 |
| **M19** | 0.001 | -0.708 | -0.789 | 0.002 | 0.002 | 0.003 | 0.096 | 0.085 | 0.181 |
| **M20** | 0.000 | -1.268 | -2.137 | 0.000 | 0.001 | 0.003 | 0.217 | 0.441 | 0.658 |
| **M21** | 0.001 | 0.551 | 2.026 | 0.004 | 0.001 | 0.010 | 0.016 | 0.156 | 0.172 |
| **M22** | 0.000 | -1.268 | -2.137 | 0.000 | 0.001 | 0.003 | 0.217 | 0.441 | 0.658 |
| **M23** | 0.064 | 0.055 | 0.030 | 0.002 | 0.001 | 0.000 | 0.039 | 0.008 | 0.048 |
| **M24** | 0.001 | -1.268 | -2.137 | 0.001 | 0.003 | 0.010 | 0.217 | 0.441 | 0.658 |
| **M25** | 0.001 | -1.268 | -2.137 | 0.002 | 0.003 | 0.014 | 0.217 | 0.441 | 0.658 |
| **M26** | **0.174** | 0.506 | 0.259 | **0.018** | **0.132** | 0.048 | **0.823** | 0.154 | 0.978 |
| **M27** | 0.079 | -0.345 | 0.060 | 0.007 | 0.028 | 0.001 | 0.459 | 0.010 | 0.469 |
| **M28** | 0.002 | -0.802 | -0.951 | 0.001 | 0.005 | 0.009 | 0.379 | 0.382 | 0.760 |
| **M29** | 0.004 | -0.766 | -0.922 | 0.002 | 0.008 | 0.016 | 0.396 | 0.412 | 0.809 |
| **M30** | 0.001 | -0.524 | -0.434 | 0.002 | 0.001 | 0.001 | 0.064 | 0.031 | 0.095 |
| **M31** | 0.002 | -0.759 | -0.843 | 0.001 | 0.004 | 0.007 | 0.329 | 0.290 | 0.619 |
| **M32** | 0.003 | -0.696 | -0.752 | 0.002 | 0.005 | 0.008 | 0.344 | 0.288 | 0.632 |
| **M33** | 0.079 | -0.102 | -0.129 | 0.003 | 0.002 | 0.006 | 0.083 | 0.096 | 0.179 |
| **M34** | 0.001 | -0.603 | -0.536 | 0.003 | 0.001 | 0.001 | 0.048 | 0.027 | 0.075 |
| **M35** | 0.005 | -0.565 | -0.315 | 0.004 | 0.005 | 0.002 | 0.153 | 0.034 | 0.187 |
| **M36** | 0.004 | -1.164 | -1.847 | 0.007 | 0.016 | 0.057 | 0.248 | 0.447 | 0.695 |
| **M37** | 0.000 | -1.268 | -2.137 | 0.000 | 0.001 | 0.003 | 0.217 | 0.441 | 0.658 |
| **M38** | 0.000 | -1.268 | -2.137 | 0.001 | 0.002 | 0.007 | 0.217 | 0.441 | 0.658 |
| **M39** | 0.001 | 0.390 | 1.705 | 0.005 | 0.001 | 0.015 | 0.014 | 0.186 | 0.200 |
| **M40** | 0.019 | 0.716 | -0.591 | 0.006 | 0.029 | 0.028 | 0.560 | 0.273 | 0.833 |
| **M41** | 0.005 | -0.360 | -0.091 | 0.004 | 0.002 | 0.000 | 0.058 | 0.003 | 0.060 |
| **M42** | 0.000 | -1.268 | -2.137 | 0.000 | 0.001 | 0.003 | 0.217 | 0.441 | 0.658 |
| **M43** | 0.000 | -1.268 | -2.137 | 0.001 | 0.002 | 0.007 | 0.217 | 0.441 | 0.658 |
| **M44** | 0.000 | -1.268 | -2.137 | 0.000 | 0.001 | 0.003 | 0.217 | 0.441 | 0.658 |
| **M45** | **0.151** | 0.367 | 0.463 | **0.017** | 0.060 | **0.134** | 0.397 | **0.453** | 0.850 |
| **M46** | 0.015 | -0.587 | -0.453 | 0.003 | 0.015 | 0.013 | 0.537 | 0.229 | 0.765 |
| **M47** | 0.015 | -0.592 | -0.539 | 0.004 | 0.015 | 0.018 | 0.488 | 0.290 | 0.777 |
| **M48** | 0.001 | -0.756 | -0.712 | 0.001 | 0.001 | 0.002 | 0.132 | 0.084 | 0.216 |
| **M49** | 0.001 | -1.268 | -2.137 | 0.001 | 0.003 | 0.010 | 0.217 | 0.441 | 0.658 |
| **M50** | 0.001 | 0.551 | 2.026 | 0.004 | 0.001 | 0.010 | 0.016 | 0.156 | 0.172 |
| **Active Total** | 1.000 |  |  | 0.238 | 1.000 | 1.000 |  |  |  |

The participants with back and/or joint pain showed highest proportion of positive responses (0.151 or 15.1%). Sinusitis (5.9%), back and/or joint pain (1.8%), dental cavity/caries (1.7%) and severe vision loss (1.6%) showed comparatively high proportion of variance. Sinusitis (dimension 1= 38.9%, dimension 2= 25.3%) loads heavily on both dimension and explains 74.9% variance (on dimension 1). Whereas back and/or joint pain (dimension 1= 13.2%, dimension 2= 4.8%) loads heavily on first dimension only with 82.3% of variance explained; and dental cavity/caries (dimension 1= 6.0%, dimension 2= 13.4%) loads heavily on second dimension with 4.3% of variance.

**Table A3.** Distance between the level of multi-morbidity and morbid conditions

| **Morbid conditions** | **Number of morbidities** | | | | | | |
| --- | --- | --- | --- | --- | --- | --- | --- |
|  | **1** | **2** | **3** | **4** | **5** | **6** | **>6** |
| **M1** | 4.106 | 3.395 | 3.118 | 3.002 | 2.536 | 2.335 | 1.826 |
| **M2** | 2.887 | 1.081 | 0.812 | 0.707 | 0.353 | **0.246** | 0.560 |
| **M2** | 3.386 | 1.526 | 1.251 | 1.131 | 0.658 | 0.496 | **0.180** |
| **M4** | **1.001** | 2.471 | 2.368 | 2.352 | 2.315 | 2.257 | 2.294 |
| **M5** | 3.352 | 0.704 | 0.467 | 0.367 | **0.320** | 0.490 | 0.980 |
| **M6** | 3.332 | 0.816 | 0.561 | 0.449 | **0.217** | 0.367 | 0.845 |
| **M7** | 5.380 | 8.466 | 8.433 | 8.442 | 8.462 | 8.407 | 8.393 |
| **M8** | 3.566 | 2.258 | 1.981 | 1.863 | 1.393 | 1.215 | 0.687 |
| **M9** | 3.509 | 0.804 | 0.595 | 0.508 | **0.431** | 0.575 | 1.015 |
| **M10** | 3.340 | 0.889 | 0.630 | 0.516 | **0.197** | 0.314 | 0.772 |
| **M11** | 3.832 | 2.886 | 2.609 | 2.492 | 2.025 | 1.845 | 1.315 |
| **M12** | 3.401 | 1.621 | 1.345 | 1.226 | 0.753 | 0.585 | **0.159** |
| **M13** | 3.348 | 1.327 | 1.053 | 0.933 | 0.463 | **0.318** | 0.322 |
| **M14** | 3.390 | 1.583 | 1.307 | 1.188 | 0.714 | 0.548 | **0.156** |
| **M15** | 3.700 | 2.600 | 2.323 | 2.205 | 1.737 | 1.557 | 1.028 |
| **M16** | 3.346 | 1.350 | 1.075 | 0.956 | 0.484 | 0.335 | **0.297** |
| **M17** | 3.354 | 0.971 | 0.709 | 0.592 | **0.210** | 0.273 | 0.695 |
| **M18** | 3.446 | 1.795 | 1.519 | 1.400 | 0.927 | 0.756 | **0.257** |
| **M19** | 3.472 | 1.955 | 1.678 | 1.560 | 1.088 | 0.912 | 0.392 |
| **M20** | 4.106 | 3.395 | 3.118 | 3.002 | 2.536 | 2.355 | 1.826 |
| **M21** | 3.919 | 1.223 | 1.477 | 1.580 | 2.018 | 2.203 | 2.724 |
| **M22** | 4.106 | 3.395 | 3.118 | 3.002 | 2.536 | 2.355 | 1.826 |
| **M23** | 2.961 | 0.860 | 0.587 | 0.477 | **0.187** | 0.234 | 0.728 |
| **M24** | 4.106 | 3.395 | 3.118 | 3.002 | 2.536 | 2.355 | 1.826 |
| **M25** | 4.106 | 3.395 | 3.118 | 3.002 | 2.536 | 2.355 | 1.826 |
| **M26** | 2.680 | 0.558 | 0.398 | **0.394** | 0.631 | 0.739 | 1.213 |
| **M27** | 3.339 | 1.069 | 0.801 | 0.683 | 0.243 | **0.218** | 0.582 |
| **M28** | 3.549 | 2.142 | 1.865 | 1.746 | 1.275 | 1.099 | 0.574 |
| **M29** | 3.515 | 2.097 | 1.820 | 1.702 | 1.231 | 1.054 | 0.529 |
| **M30** | 3.355 | 1.560 | 1.284 | 1.164 | 0.691 | 0.522 | **0.127** |
| **M31** | 3.516 | 2.029 | 1.752 | 1.633 | 1.161 | 0.986 | 0.466 |
| **M32** | 3.465 | 1.918 | 1.641 | 1.522 | 1.050 | 0.876 | 0.358 |
| **M33** | 3.043 | 1.073 | 0.797 | 0.682 | 0.248 | **0.093** | 0.505 |
| **M34** | 3.410 | 1.689 | 1.413 | 1.294 | 0.820 | 0.650 | **0.177** |
| **M35** | 3.425 | 1.493 | 1.220 | 1.100 | 0.630 | 0.478 | **0.244** |
| **M36** | 3.949 | 3.092 | 2.815 | 2.698 | 2.231 | 2.050 | 1.521 |
| **M37** | 4.106 | 3.395 | 3.118 | 3.002 | 2.536 | 2.355 | 1.826 |
| **M38** | 4.106 | 3.395 | 3.118 | 3.002 | 2.536 | 2.355 | 1.826 |
| **M39** | 3.755 | **0.895** | 1.135 | 1.233 | 1.663 | 1.847 | 2.367 |
| **M40** | 2.118 | 1.433 | 1.251 | 1.203 | 1.088 | 1.027 | 1.145 |
| **M41** | 3.298 | 1.190 | 0.916 | 0.797 | 0.327 | **0.201** | 0.430 |
| **M42** | 4.106 | 3.395 | 3.118 | 3.002 | 2.536 | 2.355 | 1.826 |
| **M43** | 4.106 | 3.395 | 3.118 | 3.002 | 2.536 | 2.355 | 1.826 |
| **M44** | 4.106 | 3.395 | 3.118 | 3.002 | 2.536 | 2.355 | 1.826 |
| **M45** | 2.910 | 0.351 | **0.152** | 0.183 | 0.587 | 0.742 | 1.261 |
| **M46** | 3.412 | 1.613 | 1.338 | 1.219 | 0.745 | 0.580 | **0.172** |
| **M47** | 3.398 | 1.685 | 1.408 | 1.289 | 0.816 | 0.645 | **0.167** |
| **M48** | 3.530 | 1.922 | 1.646 | 1.526 | 1.053 | 0.883 | 0.383 |
| **M49** | 4.106 | 3.395 | 3.118 | 3.002 | 2.536 | 2.355 | 1.826 |
| **M50** | 3.919 | 1.223 | 1.477 | 1.580 | 2.018 | 2.203 | 2.724 |

This is the tabular presentation of the bi-plot *(Figure 2)* showing the proximal distance of each morbid condition with each level of multi-morbidity. Thus, it helps to identify the closely related morbid condition(s) with each level of multi-morbidity. The estimated values of distance highlighted in bold in table A3 depicts that these conditions have the strongest correspondence both row and column wise, and was grouped accordingly (see illustration of *Figure 2)*.
